# Treatment of second to third-degree burns in a 2-day-old infant: A case report

**DOI:** 10.1016/j.ijscr.2019.07.035

**Published:** 2019-07-19

**Authors:** Thomas Ziegler, Thomas Cakl, Johannes Schauer, Dieter Pögl, Tomas Kempny

**Affiliations:** Division of Plastic and Reconstructive Surgery, Department of Surgery, Klinikum Wels-Grieskirchen, Austria

**Keywords:** TBSA, total body surface area, MSC, mesenchymal stem cell, OECD, Organisation for Economic Co-operation and Development, UCB, umbilical cord blood, HSC, hematopoietic stem cell, EPC, endothelial progenitor cell, VSEL, very small embryonic-like stem cell, EGF, epidermal growth factor, FGF, fibroblast growth factor, HDF, human dermal fibroblast, VEGF, vascular endothelial growth factor, Neonatal burn, Iatrogenic burn, Non-surgical treatment, Tissue regeneration, Mesenchymal stem cells, Infant

## Abstract

**Introduction:**

Burn injuries in newborns are particularly complex cases. Since these patients are rare, there is little experience and no existing standardized treatment.

**Presentation of case:**

This report examines a case of accidental second to third-degree burning of the heel and toes on the left foot in a new-born girl. The burns covered an estimated 1% of the total body surface area (TBSA). After an initial debridement and 32 days of non-surgical wound therapy with Adaptic® fat gauze dressings, we were able to achieve an aesthetically and functionally satisfactory result including the complete preservation of all toes. In order to eliminate a scar contracture, we carried out a Z-plasty one year later.

**Discussion:**

Modern wound treatment following the principle of less frequent dressing changes allows the burn wound to have better re-epithelialization. New findings in stem cell research indicate that the high proportion of mesenchymal stem cells (MSC) in postnatal blood is also involved in the regeneration and healing of burns. To our knowledge, this is the first case report dealing with initial non-surgical combustion therapy in a newborn.

**Conclusion:**

There is evidence that newborns have a much higher potential for wound healing than adults. Proper position in long-term immobilization of toes is important to prevent scar contracture and deformity.

## Highlights

• Therapy for high grade burns: immediate debridement, coverage with suitable dressings.
• Post-burn scar contracture-surgery: at least one year after the burn injury.
• Mobilization of Mesenchymal Stem Cells in infants is a autologous stem cell therapy.

## 1 Introduction

Burns in children have far more serious consequences than in adults, since even small burns already occupy a large percentage of the body surface [1]. In 2013, the rate of burn mortality in children aged 1–14 years was 2.5 per 100.000 worldwide, whereas in the high-income OECD area it was only 0.4 [2].

The most common burned region in children is the trunk (23.4%). The child’s foot is ranked 6th (7.7%). Children aged 0–3 years is the group (69.4%) who is most likely to suffer burns [3]. Burns in newborns, however, are rare and occur mainly in hospital setting [4].

The standard therapy for high-grade burns includes immediate debridement and coverage with suitable dressings. The task of wound dressings is to prevent transdermal fluid loss and infections and to enable re-epithelialization as well as pain-free dressing changes, simple application and cost-effectiveness [5]. We report a case of a newborn child with iatrogenic second to third-degree burn on the left foot according to the SCARE-Guidelines [6].

## 2 Presentation of case

Warming the heel for capillary blood gas control on the 2^nd^ postnatal day of a girl with a heat pad caused severe burns on the left foot. The blisters were removed at the Department of Pediatrics and treated with Jelonet ™ (Smith&Nephew plc, London, UK) a sterile Paraffin-impregnated gauze.

**Fig. 1:**
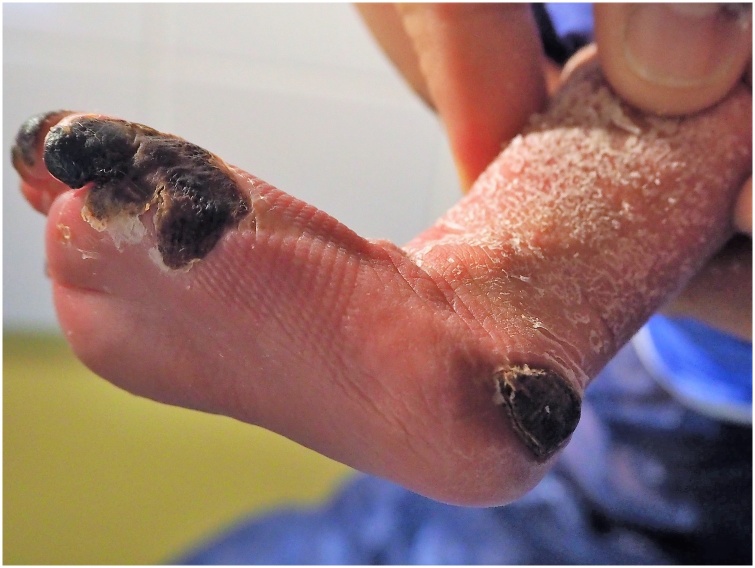
Left foot of a newborn child after superficial partial, deep partial and full thickness burn of the big toe, tip of the third, fourth and fifth toe, lateral foot margin and the heel on the sixth day of treatment with Adaptic®.

On the 4^th^ day our Division was consulted. We found superficial partial, deep partial and full thickness burning areas. The extent of the burn was about 1% of the body surface. Since the debridement was already performed, we started a conservative treatment with Adaptic® (Systagenix Wound Management Limited, Gatwick, UK) – a small mesh sized non-adhering dressing made of cellulose acetate fabric and impregnated with petrolatum emulsion. Two days later dry necroses occurred on the tip of the big toe and the 3^rd^ toe, the lateral half of the small toe, the lateral foot margin and a region of 2 × 2 cm at the heel. The extent of the necrosis in the depths could not be estimated at this point (Fig. 1). As a radical debridement would possibly have extended to the base joints of the toes and the heel bone, the necros plates were left in place.

In addition, antibiotic prophylaxis with cefuroxime was initiated. The parents were informed about the possible loss of the big and small toe.

Dressing changes with Adaptic® were carried out in 48-h intervals. To minimize scar contracture, the small toe was placed in neutral-position and underlaid with Adaptic® at the flexion fold.

The patient was discharged in good general condition on the 22^nd^ day and the subsequent dressing changes were performed in an outpatient setting.

On the 24^th^ day a black crust was removed. Underneath, new epithelialized skin appeared. From this point on, we decided to perform the dressings with dry, sterile swabs.

On the 34^th^ day, the last crust was removed. Below, rosy, well-perfused skin showed a normal capillary refill time.

**Fig. 2:**
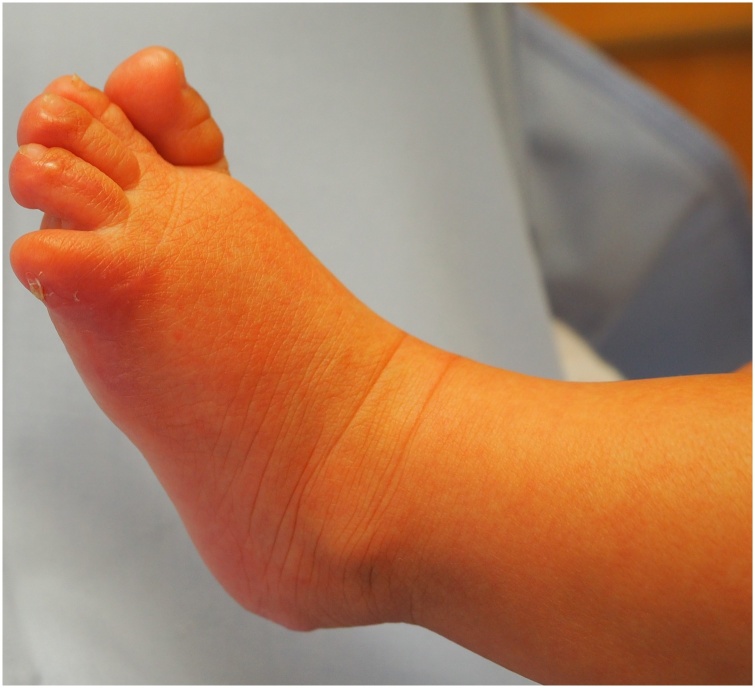
Lateral foot margin three months after burn. Lateralization of the toenail and complete re-epithelialization.

The check-up intervals were carried out on a monthly basis. On the 95^th^ day, the wounds were completely healed. Despite our efforts, the small toe showed flexion contracture compared to the right foot. The soft tissue of the small toe and its toe nail were laterally misrotated, the bone underneath, however, was palpable in an axis appropriate position (Fig. 2).

One year after the incident, a correction of the post-burn scar contracture was discussed with the parents. It was agreed to leave the nail bed in this situation for the time being and to carry out a correction at an advanced age.

**Fig. 3:**
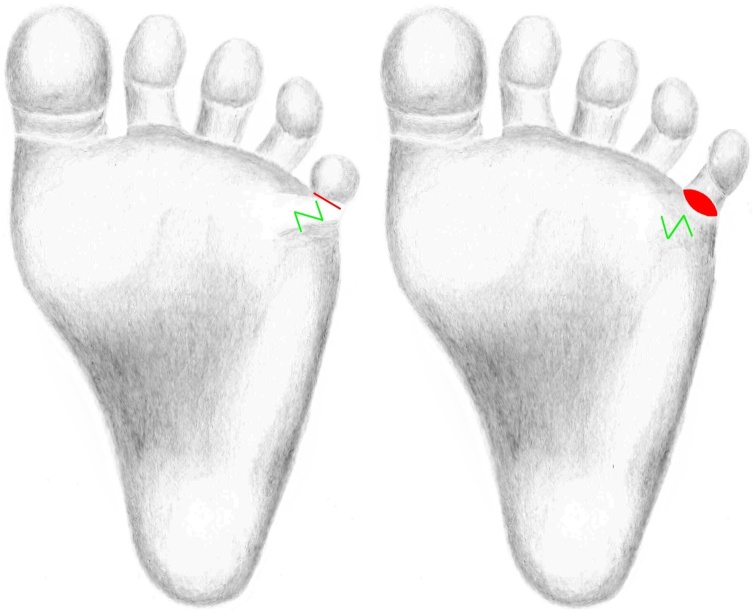
Surgical planning of the Z-plasty along the post-burn scar contracture. The marking of the incision runs along the shortened flexion fold. The post-burn scar contracture was released after performance of the Z-plasty. The full thickness skin graft was inserted into the incision defect after stretching the small toe in full extension.

We performed a Z-plasty along the contracture. The shortened flexion fold was incised and the toe was stretched in full extension. The defect size was measured in this position and at the lateral edge of the foot, outside of the instep area, a full thickness skin graft was removed and inserted into the defect (Fig. 3). At the postoperative control, the parents and we were satisfied with the aesthetic and functional outcome.

## 3 Discussion

To our knowledge, this is the first case report dealing with initial non-surgical combustion therapy in a newborn. The primary surgical approach has been reported elsewhere [4]. The extent of the burn was about 1% of the body surface. Depth and gravity of combustions can still evolve in the first 24–72 h and a re-estimation of the area is often necessary [3].

According to recent studies, it is possible to treat up to 90% of burn patients on an outpatient basis. This approach is also applicable to pediatric patients [7]. Long-term stays of burn patients bear the risk of infection with multidrug-resistant microorganisms and increase the overall risk of the patient. Yet, a stationary stay allows a more effective immobilization [5].

Excessive burns of the foot can damage tendons or joints and often require the use of microvascular or pedicled flaps. There are many innovative techniques in the surgical treatment of burns [8]. Since the number of burn victims is high, especially in developing countries, standardized and cost-effective therapies should be made available [9]. As surgical therapy bears the risk of possible limitations of functionalities crucial for the development of the foot, we favored an initial non-surgical approach. Surgery for post-burn scar contractures should be performed at least one year after the burn injury and not during the active phase of wound healing [10].

Modern wound therapy of burns is carried out according to the principle of less frequent dressing changes. This allows the burn wound to have a better re-epithelialization [5]. We preferred fatty gauze dressing over the usual wound treatment with coated foam, as this allowed us to better cover the surface structures of the child's foot. Adaptic® consists of a network of cellulose acetate fibers coated with a petrolatum emulsion, contains surfactant and reduces the surface tension and thus allows easy passage of exudate. We have decided to switch from Jelonet ™ to Adaptic®, as it is easier to remove and significantly less pain is associated with the removal of Adaptic®. Adaptic® requires less soaking and causes less maceration than Jelonet ™ [11].

After removal of the necroses and visualization of the underlying juvenile keratinocytes, we switched from Adaptic® to dry, sterile swabs. It is believed that direct contact with fatty wound dressings may adversely affect cell growth and survival of keratinocytes in the early wound healing phase. Keratinocytes that were in contact with Adaptic® showed increased mortality, a decreased division rate, and changes in cell morphology, increased LDH liberation, and increased cell damage in an in vitro study [12].

There is evidence that newborns have a much higher potential for wound healing than adults. Umbilical cord blood (UCB) and thus the blood of the newborn is rich in stem cells such as mesenchymal stem cells (MSCs), hematopoietic stem cells (HSCs), endothelial progenitor cells (EPCs) and very small embryonic-like stem cells (VSELs). They could play a role in tissue repair after birth [13]. Stem cells are immature progenitor cells that are capable of self-renewal of various tissues [14].

MSCs secrete cytokines and numerous growth factors like epidermal growth factor (EGF) and fibroblast growth factor (FGF), which both play an integral role in skin rejuvenation and wound healing. This is accomplished by collagen synthesis of human dermal fibroblasts (HDFs) [14]. Umbilical Cord Blood Derived Mesenchymal Stem Cells (UCB-MSCs) express higher amounts of wound healing factors than other MSCs [15].

The presence of these cells in the neonatal blood circulation results from hypoxia and the rise of cytokines induced by multiple small tissue injuries during birth. This mobilization of stem cells is regarded as a autologous physiological stem cell therapy [13].

It was observed that the concentration of very small pluripotent embryotic stem cells in the peripheral blood increases during organ and tissue damage [16] as well as to burns [17]. The use of mesenchymal stem cells for the therapy of radiation induced burns has already been described [18]. Positive effects of mesenchymal stem cells on the regeneration of severe burns had been confirmed in an animal experiment [19]. These stem cells express the surface markers CD44, 73, 90 and 105. The antifibrotic wound healing factor HGF, which is involved in scarless wound healing, is particularly expressed in UCB-MSCs. These gene expression profiles indicate that UCB-MSCs could be a stem cell source for scarless wound healing. It can be assumed that neonatal blood has the same potential [20].

The skin is the organ with the highest number of stem cells and therefore capable of extraordinary regeneration after injuries or burns [17]. Apart from MSCs other stem cells, such as epithelial stem cells, adipose-derived stem cells and fibroblasts, might play important roles in tissue regeneration.

## 4 Conclusion

We assume that apart from the primary debridement, it was mainly the conservative approach under sterile conditions, the antibiotic prophylaxis to prevent bacterial infections and the increasingly well-understood effect of stem cells in the blood of newborns that finally led to this satisfactory result. The functional result, however, could only be improved by surgical intervention. It showed that in case of long-term immobilization of toes, more attention should be paid to the proper position to prevent scar contracture and deformity.

## Declaration of Competing Interest

None.

## Sources of funding

None.

## Ethical approval

This is a case report in which no research has been done. The name of the patient is not disclosed. Ethical approval was not required.

## Consent

Written informed consent was obtained from the patients parents for publication of this case report and accompanying images. A copy of the written consent is available for review by the Editor-in-Chief of this journal on request.

## Author contribution

Thomas Ziegler: Study design, creation of graphics, literature research, selection of studies, conception and preparation of the manuscript, drafting of the article, proofreading and revising for important content. Final approval of the submitted version.

Thomas Cakl: Creation of graphics conception and preparation of the manuscript, proofreading and revising for important content. Final approval of the submitted version.

Johannes Schauer: Conception and preparation of the manuscript, proofreading and revising for important content. Final approval of the submitted version.

Dieter Pögl: Conception and preparation of the manuscript, proofreading and revising for important content. Final approval of the submitted version.

Tomas Kempny: Conception and preparation of the manuscript, proofreading and revising for important content. Final approval of the submitted version.

## Registration of research studies

Not applicable.

## Guarantor

Thomas Ziegler M.D.

## Provenance and peer review

Not commissioned, externally peer-reviewed.

## References

[1] Celko A.M., Grivna M., Danova J., Barss P. (2009). Severe childhood burns in the Czech Republic: risk factors and prevention. Bull. World Health Organ..

[2] Sengoelge M., El-Khatib Z., Laflamme L. (2017). The global burden of child burn injuries in light of country level economic development and income inequality. Prev. Med. Rep..

[3] Clifton L.J., Chong L.W., Stewart K. (2015). Identification of factors that predict outpatient utilisation of a plastic dressing clinic. A retrospective review of 287 paediatric burn cases. Burns.

[4] Abboud L., Ghanimeh G. (2017). Thermal burn in a 30-minute-old newborn: report on the youngest patient with iatrogenic burn injury. Ann. Burns Fire Disasters.

[5] Hundeshagen G., Collins V.N., Wurzer P. (2018). A prospective, randomized, controlled trial comparing the outpatient treatment of pediatric and adult partial-thickness burns with suprathel or mepilex ag. J. Burn Care Res..

[6] Agha R.A., Borrelli M.R., Farwana R. (2018). The SCARE 2018 statement: updating consensus Surgical CAse REport (SCARE) guidelines. Int. J. Surg..

[7] Brown M., Coffee T., Adenuga P., Yowler C.J. (2014). Outcomes of outpatient management of pediatric burns. J. Burn Care Res..

[8] Wang F., Liu S., Qiu L. (2015). Superthin abdominal wall glove-like flap combined with vacuum-assisted closure therapy for soft tissue reconstruction in severely burned hands or with infection. Ann. Plast. Surg..

[9] Amouzou K.S., El Harti A., Kouevi-Koko T.E., Abalo A., Dossim A. (2016). Treatment of an acute deep hand burn in a lowincome country with no available microsurgery: a case report. Ann. Burns Fire Disasters.

[10] Goel A., Shrivastava P. (2010). Post-burn scars and scar contractures. Indian J. Plast. Surg..

[11] Terrill P.J., Varughese G. (2000). A comparison of three primary non-adherent dressings applied to hand surgery wounds. J. Wound Care.

[12] Esteban-Vives R., Young M.T., Ziembicki J., Corcos A., Gerlach J.C. (2016). Effects of wound dressings on cultured primary keratinocytes. Burns.

[13] Sielatycka K., Poniewierska-Baran A., Nurek K., Torbe A., Ratajczak M.Z. (2017). Novel view on umbilical cord blood and maternal peripheral blood-an evidence for an increase in the number of circulating stem cells on both sides of the fetal-maternal circulation barrier. Stem Cell Rev..

[14] Kim Y.J., Yoo S.M., Park H.H. (2017). Exosomes derived from human umbilical cord blood mesenchymal stem cells stimulates rejuvenation of human skin. Biochem. Biophys. Res. Commun..

[15] Kim J., Lee J.H., Yeo S.M., Chung H.M., Chae J.I. (2014). Stem cell recruitment factors secreted from cord blood-derived stem cells that are not secreted from mature endothelial cells enhance wound healing. In Vitro Cell. Dev. Biol. Anim..

[16] Ratajczak M.Z., Liu R., Marlicz W. (2011). Identification of very small embryonic/epiblast-like stem cells (VSELs) circulating in peripheral blood during organ/tissue injuries. Methods Cell Biol..

[17] Drukala J., Paczkowska E., Kucia M. (2012). Stem cells, including a population of very small embryonic-like stem cells, are mobilized into peripheral blood in patients after skin burn injury. Stem Cell Rev..

[18] Leclerc T., Thepenier C., Jault P. (2011). Cell therapy of burns. Cell Prolif..

[19] Liu L., Yu Y., Hou Y. (2014). Human umbilical cord mesenchymal stem cells transplantation promotes cutaneous wound healing of severe burned rats. PLoS One.

[20] Doi H., Kitajima Y., Luo L. (2016). Potency of umbilical cord blood- and Wharton’s jelly-derived mesenchymal stem cells for scarless wound healing. Sci. Rep..

